# Predictors of response and survival in cemiplimab–treated cutaneous squamous cell carcinoma: multicenter real-world evidence from Germany

**DOI:** 10.1007/s00432-026-06423-x

**Published:** 2026-02-25

**Authors:** Thilo Gambichler, Josefine Brune, Jonas Rüth, Nessr Abu Rached, Stefanie Boms, Sera S. Weyer-Fahlbusch, Alexander Kreuter, Julia Hyun, Jürgen C. Becker, Laura Susok

**Affiliations:** 1 Department of Dermatology, Dortmund Hospital gGmbH, University Witten/Herdecke, Dortmund, Germany; 2 Department of Dermatology, Skin Cancer Center, Ruhr-University Bochum, Bochum, Germany; 3 Department of Dermatology, Christian Hospital Unna, Unna, Germany; 4 Department of Dermatology, Bielefeld University, Medical School and University Medical Center OWL, Klinikum Bielefeld Rosenhöhe, Bielefeld, Germany; 5 Department of Dermatology, Venereology, and Allergology, HELIOS St. Elisabeth Hospital Oberhausen, Oberhausen, Germany; 6 Department of Dermatology, Venereology, and Allergology, Helios St. Johannes Hospital Duisburg, Duisburg, Germany; 7 Departments of Translational Skin Cancer Research and Dermatology, University Hospital Essen, Essen, Germany; German Cancer Consortium (DKTK), Partner Site Essen/Düsseldorf and German Cancer Research Center (DKFZ), Heidelberg, Germany

**Keywords:** Cemiplimab, Pembrolizumab, Inflammatory biomarkers, Lymphocyte-monocyte ratio, LMR, NLR, BMI, Immunotherapy, PD-1 inhibitor

## Abstract

**Purpose:**

To assess the association of systemic immune-inflammation biomarkers (SIIBs) and other clinical parameters with objective response rate (ORR), progression-free survival (PFS), overall survival (OS), disease-specific survival (DSS), and immune-related adverse events (irAEs) in patients with advanced cSCC treated with cemiplimab, and to compare baseline SIIBs levels between early-stage and advanced-stage disease.

**Methods:**

A retrospective multicenter cohort of 110 immunocompetent advanced cSCC patients treated with cemiplimab was analysed. ORR was assessed using logistic regression; PFS and OS were evaluated using Cox models, and DSS using cause-specific hazards. ROC analyses assessed biomarker discrimination. Baseline SIIBs (LMR, NLR, SIRI) were compared between early-stage (AJCC I/II, non-ICI cohort, *n* = 59) and advanced-stage disease. Tumor characteristics, body mass index (BMI), and Charlson comorbidity index were evaluated.

**Results:**

Among 110 patients, 79 (71.8%) achieved an objective response. Baseline LMR showed modest discrimination for ORR (AUC 0.64, 95% CI 0.53–0.75; *p* = 0.015) but did not retain statistical significance after adjustment for baseline clinical covariates (OR 1.35, 95% CI 0.95–1.91; *p* = 0.096). Higher BMI was associated with improved PFS (HR 0.94 per kg/m^2^, 95% CI 0.89–1.00; *p* = 0.035) and showed a borderline association with OS (HR 0.92 per kg/m^2^, 95% CI 0.85–1.00; *p* = 0.051). AJCC stage IV strongly predicted DSS (HR 14.03, 95% CI 1.80–109.67; *p* = 0.012). Baseline LMR was higher in early-stage than in advanced-stage disease (Hodges-Lehmann difference 0.43; *p* = 0.011), whereas NLR did not differ significantly between stage groups; SIRI was modestly higher in advanced-stage disease (*p* = 0.029).

**Conclusions:**

In immunocompetent patients with advanced cSCC receiving PD-1 inhibition, BMI was prognostic for survival and AJCC stage remained the key driver of cSCC-specific mortality. Baseline LMR showed a modest association with response and differed between early- and advanced-stage disease, whereas other SIIBs were not consistently linked to tumor progression. Prospective validation is warranted.

**Supplementary Information:**

The online version contains supplementary material available at 10.1007/s00432-026-06423-x.

## Introduction

 Cutaneous squamous cell carcinoma (cSCC) is one of the most common skin cancers, with rising incidence particularly among elderly and immunosuppressed populations. While most cases are cured surgically, a clinically relevant subset presents with locally advanced or metastatic disease associated with substantial morbidity and mortality. Immune checkpoint inhibitors (ICIs), particularly PD-1 inhibitors such as cemiplimab, nivolumab, and pembrolizumab, have transformed the management of advanced cSCC and other skin cancers, achieving objective response rates of 45–60% and providing durable disease control (Leiter et al. 2023; Jiang et al. 2024; Strum et al. 2024; Migden et al. 2020; Ríos-Viñuela et al. 2022). Despite these advances, validated predictive biomarkers for treatment selection remain absent.

Systemic immune-inflammatory biomarkers (SIIB) derived from routine blood counts have emerged as promising tools across multiple malignancies, including melanoma, Merkel cell carcinoma, and colorectal cancer (Gambichler et al. 2022, 2022; Susok et al. 2022; Yang et al. 2025; Ou et al. 2024). These markers reflect the balance between immunosuppressive myeloid components and lymphocyte-mediated antitumor immunity. For example, a high neutrophil-to-lymphocyte ratio (NLR) has been linked to tumor progression, impaired immune surveillance, and poor outcomes in several cancers (Arianmanesh et al. 2025). In cSCC specifically, elevated NLR has been associated with advanced stage, sentinel lymph node positivity, and reduced disease-specific survival (DSS) (Maeda et al. 2022). Strippoli et al. (2021) showed that low NLR and platelet-to-lymphocyte ratio were associated with better response to cemiplimab therapy of advanced cSCC. Accordingly, in a cohort of patients with NSCLC treated with nivolumab in routine practice, pretreatment NLR ≥ 5 was associated with inferior outcomes (Bagley et al. 2017). Conversely, the lymphocyte-to-monocyte ratio (LMR) captures the interplay between effective cytotoxic immunity and monocyte-driven tumor-supportive inflammation and has been associated with improved survival in melanoma and other malignancies (Iacono et al. 2019). However, data on LMR in cSCC, particularly under PD-1 inhibition, remain extremely limited. We found only one study in the literature comparing LMR in the context of sun exposure of patients with cSCC and basosquamous carcinoma (Jairath et al. 2020).

Given the absence of established biomarkers and the biological plausibility of inflammation-derived metrics, we aimed to evaluate LMR as our primary inflammatory biomarker, alongside exploratory analyses of NLR and systemic inflammation response index (SIRI), in a real-world multicenter cohort of patients with advanced cSCC treated with the PD-1 inhibitor cemiplimab.

## Methods

### Patients and in- and exclusion criteria

Patients with histologically confirmed advanced cSCC who received at least two cycles of ICI therapy with cemiplimab were eligible. Based on tumor board recommendation, all patients had an indication for cemiplimab treatment per national guideline (Leiter et al. 2023). Baseline staging comprised lymph‑node ultrasound, thoraco‑abdominal computed tomography, and cranial magnetic resonance imaging. Clinical work‑up, treatment, and follow‑up were performed according to national guideline (Leiter et al. 2023).

Baseline complete blood counts, including differential leukocyte parameters, had to be available within 14 days prior to treatment initiation. Tumor response had to be assessable according to RECIST 1.1 or equivalent clinical criteria. Follow-up data for at least one time-to-event endpoint (progression, death, or cause-specific death) were required for inclusion in the corresponding survival analyses. Patients with incomplete outcome information for a given endpoint were excluded only from that specific analysis. Moreover, clinical data including tumor parameters, body mass index (BMI, and Charlson comorbidity index (CCI) was evaluated.

Patients were excluded if baseline blood counts were missing or obtained during acute infection, systemic inflammatory conditions, or antibiotic therapy within 14 days before treatment start. Additional exclusion criteria included hematologic malignancies known to alter leukocyte composition and systemic immunosuppression (e.g., solid-organ transplantation, hematologic/immunologic conditions requiring immunosuppressive therapy, or chronic corticosteroids > 10 mg prednisolone equivalent daily). Patients who discontinued PD-1 inhibition after a single cycle, lacked follow-up imaging, or had incomplete response evaluation were excluded from efficacy analyses.

The primary endpoint was the objective response rate (ORR), defined as complete or partial response per RECIST 1.1 or clinically equivalent criteria. Secondary endpoints included progression-free survival (PFS), overall survival (OS), DSS, and immune-related adverse events (irAEs). PFS was defined as the time from PD-1 initiation to progression or death from any cause. OS was defined as the time from treatment initiation to death from any cause. DSS was defined as death attributable to cSCC, with non-cSCC deaths treated as competing events.

### Blood-based systemic immune-inflammation biomarkers

Baseline SIIBs, including the NLR (neutrophils/lymphocytes), the LMR (lymphocytes/monocytes), and the SIRI (neutrophils × monocytes / lymphocytes), were obtained within 14 days prior to PD-1 initiation (Gambichler et al. 2022). Follow-up blood counts at approximately three months were available for most patients; however, biomarker levels did not show relevant systematic changes compared with baseline and were therefore not incorporated as dynamic covariates. LMR was defined as the principal biomarker of interest, whereas NLR and SIRI were analysed as exploratory variables. All biomarkers were evaluated as continuous variables, with exploratory dichotomisation using ROC-derived thresholds.

### Statistics

As this was a retrospective observational study, no prospective sample size calculation was performed. All eligible immunocompetent patients treated during the study period were included (*n* = 110). The number of events (PFS: 62; OS: 34; DSS: 11) permitted parsimonious time-to-event modelling to reduce overfitting. Where appropriate, we used the paired Wilcoxon test, Mann-Whitney test for and independent data, Spearman rank correlation procedure, and ROC curves. Logistic regression was used to assess associations with ORR. Multivariable ORR models were restricted to baseline variables (age, sex, AJCC stage, tumor differentiation and selected SIIBs); the number of administered ICI cycles was considered a post-baseline variable and was not used as a predictor. Kaplan-Meier curves were compared using the log-rank test. Cox proportional hazards models were used for PFS and OS; DSS was analysed using cause-specific Cox regression with deaths from other causes treated as censored events. For early-stage vs. advanced-stage comparisons, Hodges-Lehmann median differences and Mann-Whitney tests were used.

## Results

### Patient cohort and baseline characteristics

**Fig. 1:**
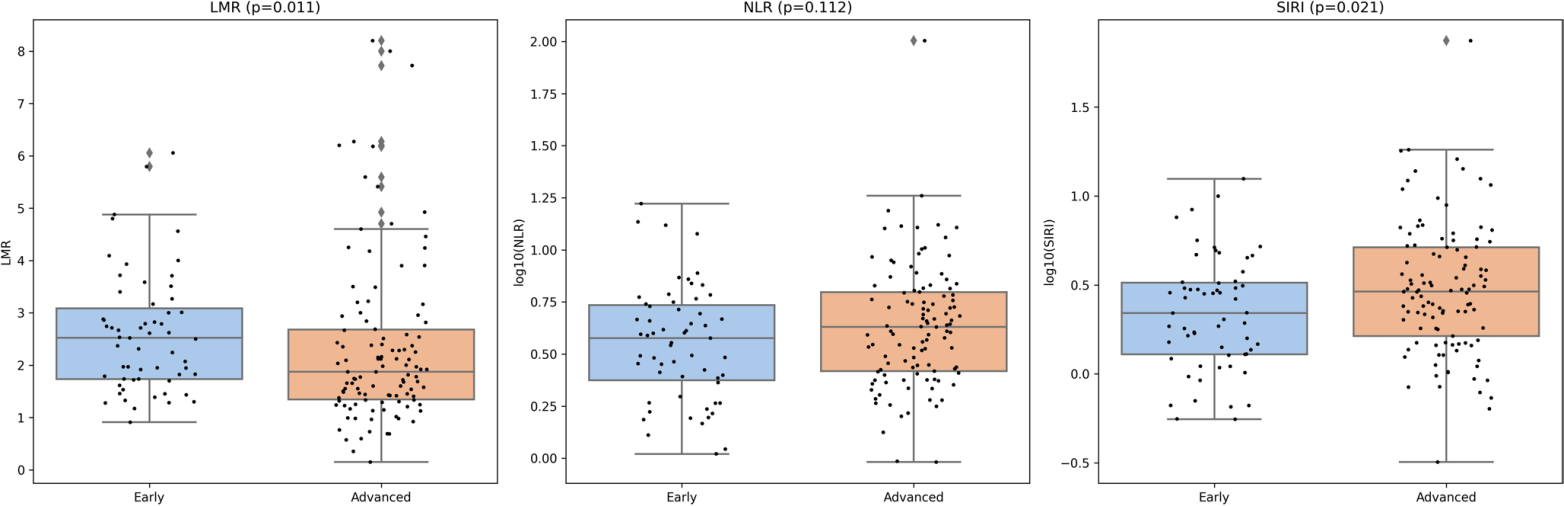
Comparison of baseline systemic immune-inflammatory biomarkers (SIIBs) between early-stage and advanced-stage cutaneous squamous cell carcinoma (cSCC). Panel A shows lymphocyte-to-monocyte ratio (LMR) on a linear scale; panels B and C depict neutrophil-to-lymphocyte ratio (NLR) and systemic inflammation response index (SIRI) on a log10 scale due to skewed distributions. Early-stage patients (non-ICI cohort) demonstrated higher LMR values, whereas SIRI values were higher in advanced-stage patients treated with PD-1 blockade. NLR did not differ significantly between groups. Boxplots display medians and interquartile ranges; individual points represent patient-level values (jittered for visibility). P-values are based on two-sided Mann–Whitney U tests

A total of 110 immunocompetent patients with advanced cSCC treated with PD-1 inhibition were included. Median age was 84 years (range 39–99) and 74/110 (67.3%) were male. Baseline inflammatory markers showed a right-skewed distribution (median [IQR]: NLR 4.17 [2.60–6.26], LMR 1.87 [1.34–2.68], SIRI 2.90 [1.56–5.10]). Follow-up blood counts at approximately 3 months were available for 100/110 patients and biomarker levels did not differ meaningfully from baseline (paired Wilcoxon, Table 1). In the early-stage comparator cohort (AJCC I/II, *n* = 59), baseline LMR was higher than in the advanced cohort (Hodges-Lehmann difference 0.43; *p* = 0.011), whereas NLR showed no significant stage-dependent difference (*p* = 0.139); SIRI was modestly lower in early-stage disease (Fig. 1, *p* = 0.029). SIRI was strongly correlated with NLR (Spearman, *r* ≈ 0.88) and inversely correlated with LMR (Spearman, *r* ≈ − 0.88). Baseline characteristics stratified by baseline LMR, SIRI, and NLR are provided in supplementary Table S1.

**Table 1:** Characteristics of 110 patients with advanced cutaneous squamous cell carcinoma treated with Cemiplimab

Parameters	Data
Age in years, median, range	84, 39–99
Sex (f, m)	36 (32.7%), 74 (67.3%)
BMI (mean ± SD)	25.6 ± 4.7 (median 24.8)
Charlson Comorbidity Index (mean ± SD)	8.2 ± 2.6 (median 8.0)
Primary tumor location (head/neck, upper limbs, lower limbs, trunk, genital)	77 (70.0%), 17 (15.5%), 6 (5.5%), 4 (3.6%), 6 (5.5%)
Lower lip / ear involvement (no, yes)	86 (78.2%), 24 (21.8%)
Tumor differentiation ≥ G3 (no, yes)	89 (80.9%), 21 (19.1%)
Breslow thickness > 6 mm (no, yes)	78 (70.9%), 32 (29.1%)
Horizontal diameter > 2 cm (no, yes)	45 (40.9%), 65 (59.1%)
Perineural invasion/desmoplasia (no, yes)	85 (77.3%), 25 (22.7%)
AJCC stage (II, III, IV)	12 (10.9%), 53 (48.2%), 45 (40.9%)
Radiotherapy to primary tumor (no, yes)	67 (60.9%), 43 (39.1%)
Number of ICI cycles (mean ± SD)	10.6 ± 9.8
LMR (median, range)	Baseline: 1.9 (0.15–8.20); 3 months: 1.9 (0.20–7.53), *p* = 0.28
NLR (median, range)	Baseline: 4.3 (0.96-100.92); 3 months: 3.8 (1.06–63.62), *p* = 0.39
SIRI (median, range)	Baseline: 2.9 (0.32–74.68); 3 months: 2.65 (0.48–60.94), *p* = 0.18

### Objective response rate

**Fig. 2:**
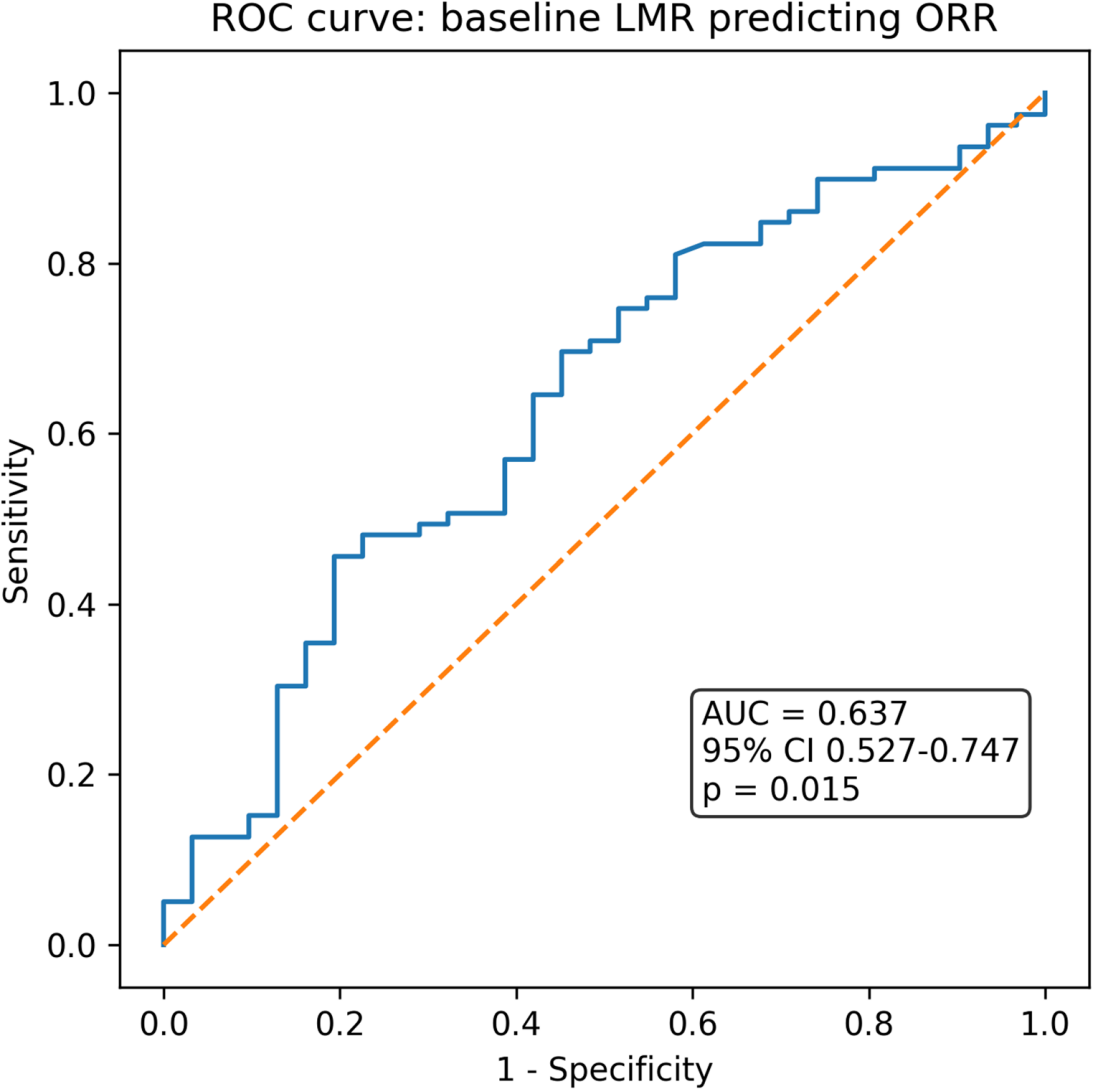
Receiver operating characteristic (ROC) curve for baseline lymphocyte-to-monocyte ratio (LMR) predicting objective response (ORR). LMR showed modest discriminative ability with an area under the curve (AUC) of 0.64 (95% CI 0.53–0.75; *p* = 0.015), indicating that higher LMR values were associated with an increased probability of response to PD-1 inhibition

Among the 110 patients, 79 (71.8%) achieved an objective response. Responders received more ICI cycles than non-responders (median 9 vs. 3 cycles; *p* < 0.001), just reflecting reverse causality. Baseline LMR was higher in responders than non-responders (median 2.00 vs. 1.54; *p* = 0.026), yielding modest discrimination (AUC 0.64, 95% CI 0.53–0.75; *p* = 0.015; Fig. 2). In multivariable logistic regression adjusting for age, sex, AJCC stage IV, and tumor differentiation ≥ G3, baseline LMR did not retain statistical significance (OR 1.35, 95% CI 0.95–1.91; *p* = 0.096).

### Progression-free survival

**Fig. 3:**
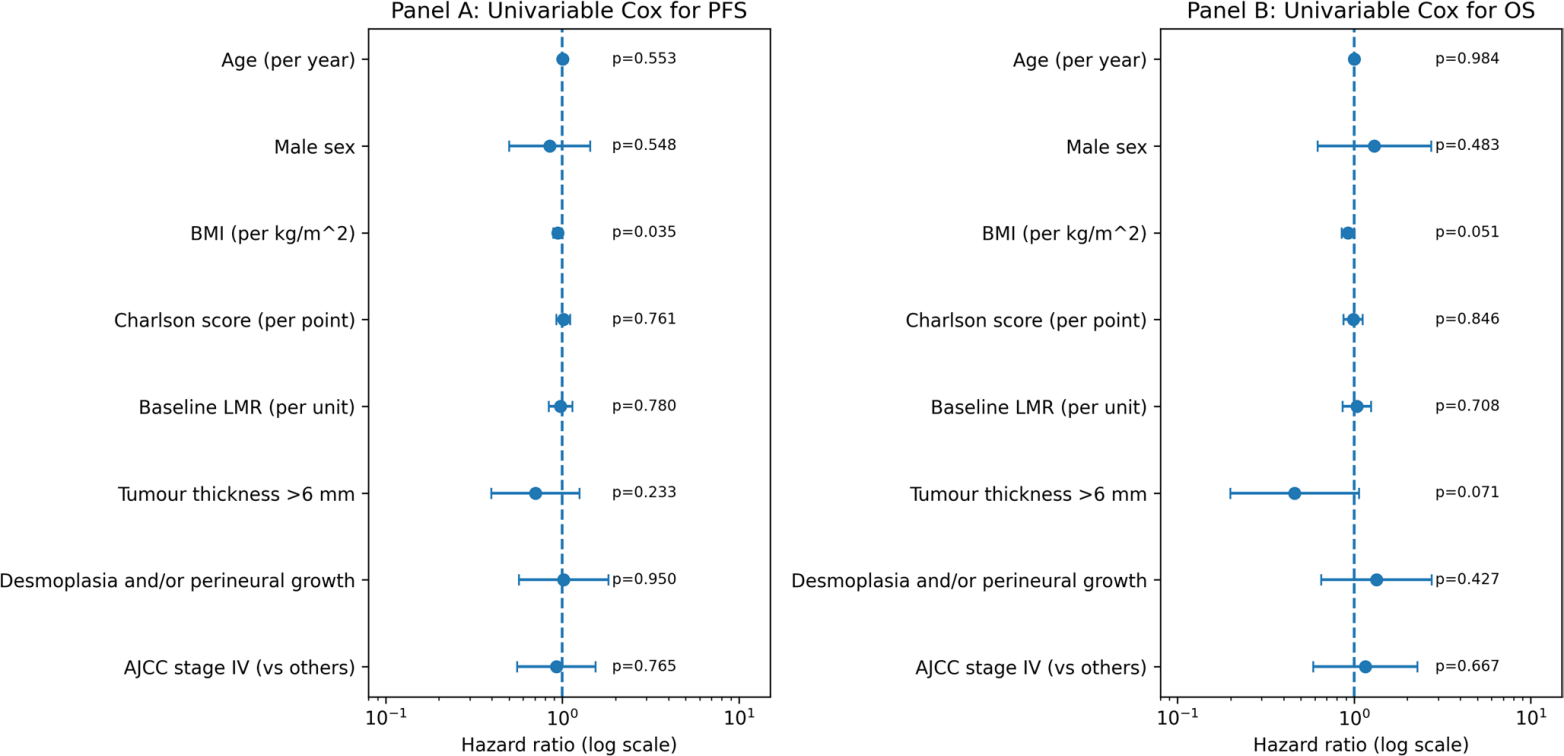
Panel A: Forest plot showing univariable Cox hazard ratios for progression-free survival (PFS) according to baseline clinical and inflammatory parameters. BMI was the only variable significantly associated with PFS (HR 0.94, 95% CI 0.89–1.00; *p* = 0.035), indicating a modest protective effect. All other parameters, including age, sex, CCI score, LMR, tumor thickness > 6 mm, perineural invasion, and AJCC stage (IV vs. III), were not significantly associated with PFS. Hazard ratios are plotted on a logarithmic scale; circles represent point estimates and horizontal bars denote 95% confidence intervals. Panel B: Forest plot showing univariable Cox hazard ratios for overall survival (OS). Higher BMI showed a borderline protective association with OS (HR 0.92, 95% CI 0.85–1.00; *p* = 0.051). Interestingly, tumor thickness  > 6 mm showed a borderline association with reduced mortality risk (HR 0.46, 95% CI 0.20–1.03; *p* = 0.071). Other variables did not demonstrate statistically significant associations with OS

**Fig. 4:**
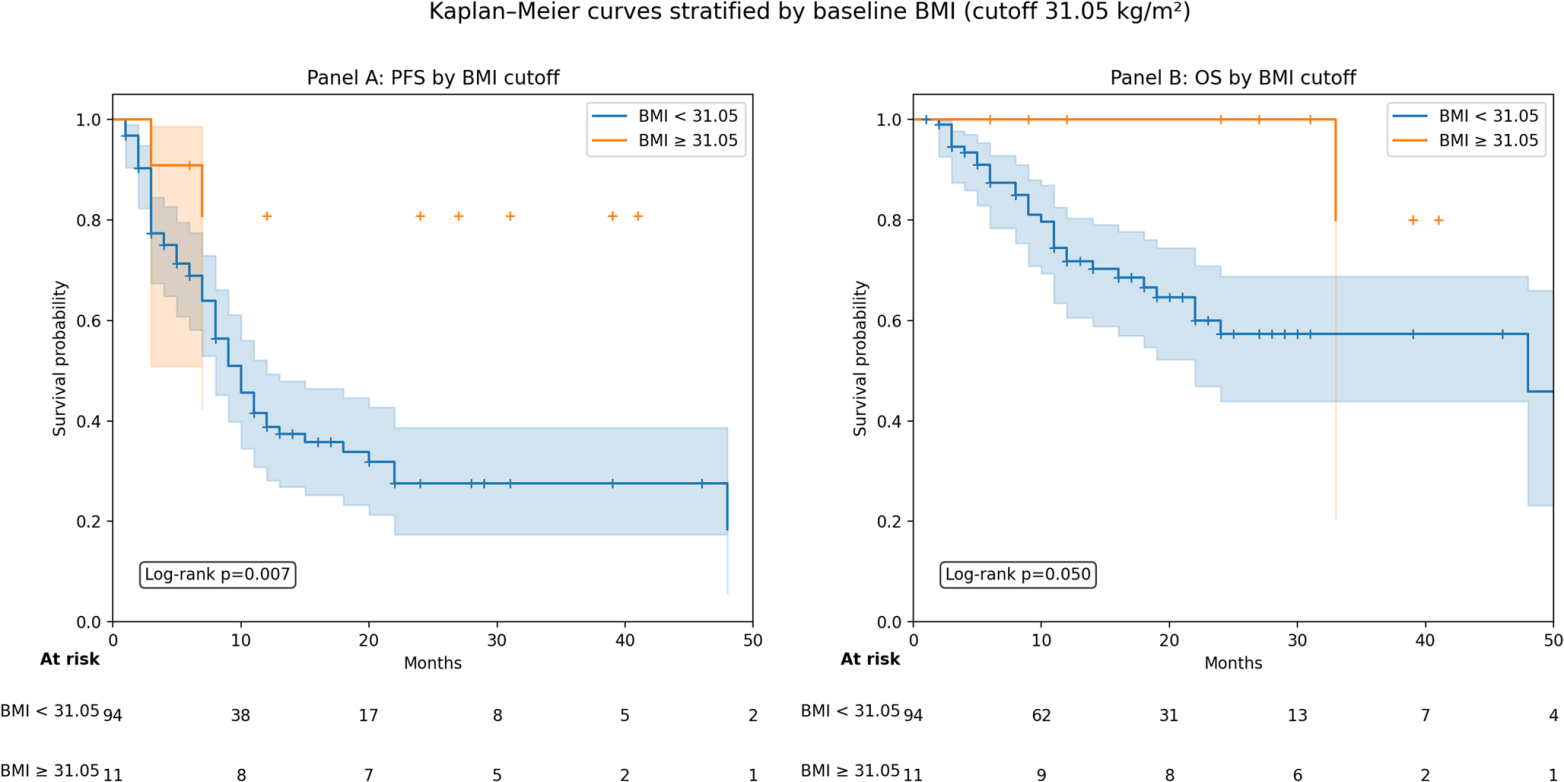
Kaplan-Meier estimates of progression-free survival (PFS) and overall survival (OS) stratified by baseline BMI (cutoff 31.05 kg/m^2^; exploratory ROC-derived cutoff). Panel A: PFS stratified by BMI. Median PFS was not reached in the high-BMI group ( > = 31.05 kg/m^2^; *n* = 11), whereas it was 10 months in the low-BMI group (< 31.05 kg/m^2^; *n* = 94). The log-rank test showed a significant difference between groups (*p* = 0.007). In univariable Cox regression, low BMI was associated with an increased risk of progression (HR 5.36, 95% CI 1.33–21.67; *p* = 0.020). Panel B: OS stratified by the same BMI cutoff. Median OS was not reached in the high-BMI group and was 48 months in the low-BMI group; the log-rank test was borderline (*p* = 0.050). In univariable Cox regression, high BMI showed a non-significant protective association (HR 0.17, 95% CI 0.02–1.28; *p* = 0.086). Shaded areas denote 95% confidence intervals; tick marks indicate censored observations

Among 110 patients, 62 (56.4%) experienced progression. Median PFS was 8 months (IQR 3-16.8). In forward-selected Cox regression (based on 105 patients with available BMI), BMI was the only retained covariate (HR 0.94 per kg/m^2^, 95% CI 0.89–1.00; *p* = 0.035; C-index 0.561). SIIBs were not associated with PFS (Figs. 3 and 4).

### Overall survival

A total of 34/110 patients (30.9%) died during follow-up. Median OS was 52 months. In univariable Cox regression (*n* = 105 with available BMI), BMI showed a borderline protective association with OS (HR 0.92 per kg/m^2^, 95% CI 0.85–1.00; *p* = 0.051; C-index 0.598). No SIIB was significantly associated with OS.

### Disease-specific survival

Eleven cSCC-specific deaths were documented (10.0%). In the cause-specific Cox model, AJCC stage IV was the dominant predictor of DSS (HR 14.03, 95% CI 1.80–109.67; *p* = 0.012).

### Immune-related adverse events

Twenty-eight patients (25.5%) experienced irAEs. Baseline SIIBs did not show a robust association with irAE occurrence; baseline NLR showed only weak, non-significant discrimination (AUC 0.60; *p* = 0.105).

## Discussion

ICIs have become the standard of care for advanced cSCC, demonstrating high activity and durable responses in clinical trials and real-world cohorts (Strum et al. 2024; Migden et al. 2020; Ríos-Viñuela et al. 2022; Grigore et al. 2024; Xiao et al. 2025; Ksienski et al. 2024; Samaran et al. 2023). Despite these advances, however, reliable and widely applicable biomarkers to guide treatment decisions remain lacking. In this multicenter real-world study, we evaluated more or less commonly used SIIBs (NLR, LMR, SIRI), alongside BMI, CCI, and AJCC staging, in order to characterize predictors of response and survival under PD-1 inhibition.

Among all evaluated biomarkers, LMR showed a modest association with ORR, which is biologically plausible given that LMR reflects the balance between lymphocyte-mediated antitumor immunity and monocyte-driven immunosuppressive pathways. Higher LMR has been linked to improved outcomes in melanoma, glioma, and gastric, oesophageal, and pancreatic cancer (Di Raimondo et al. 2022; Aoyama et al. 2024, 2024; Wang et al. 2023; Xue et al. 2017), and our findings support the relevance of this axis in cSCC. Notably, early-stage cSCC patients demonstrated significantly higher LMR values than advanced-stage patients, reinforcing the concept that tumor progression is accompanied by a shift toward monocyte-dominant systemic inflammation, consistent with previous reports identifying systemic inflammation as a hallmark of advanced cSCC biology (Samaran et al. 2023). However, after adjustment for baseline clinical covariates, including AJCC stage and tumor differentiation, LMR did not retain statistical significance as an independent predictor of ORR, suggesting that it may reflect host immune fitness rather than exert a direct tumor-specific predictive effect. Moreover, LMR and the other SIIBs were not associated with PFS or OS within the advanced-stage cohort, indicating that while these markers capture stage-related immune alterations, they have limited utility for predicting progression dynamics under PD-1 blockade.

Baseline SIIBs were strongly intercorrelated, consistent with overlapping leukocyte components and explaining the limited incremental value of SIRI over simpler indices. Contrary to earlier studies in surgically managed or mixed-stage cSCC populations (Grigore et al. 2024; Xiao et al. 2025), baseline NLR and SIRI did not predict ORR, PFS, OS, or DSS in our immunocompetent advanced-stage cohort. Similar discrepancies have been described in other real-world cemiplimab analyses, where NLR was variably associated with survival or clinical deterioration (Ksienski et al. 2024). These divergent findings may relate to differences in population age, comorbidity burden, and tumor biology; patients with advanced cSCC treated with ICIs often represent an elderly, immunosenescent, yet immunotherapy-responsive population (Ksienski et al. 2024), in whom NLR and related indices may be more strongly influenced by chronic host factors than by tumor-associated inflammation. Nevertheless, NLR did demonstrate a modest association with irAEs in our cohort, consistent with observations in other malignancies suggesting that patients with more immunoreactive baseline inflammatory profiles may be more susceptible to immune toxicity (Gambichler et al. 2022). In addition to biomarker findings, treatment exposure differed markedly between clinical responders and non-responders (median 9 vs. 4 cycles), a pattern that has also been reported in other real-world cemiplimab cohorts (Xiao et al. 2025; Ksienski et al. 2024). This asymmetry most likely reflects reverse causality - longer treatment duration as a consequence of response - rather than a causal effect of cycle number on tumor regression, underscoring the importance of interpreting treatment duration as an outcome-dependent parameter rather than an independent predictor.

BMI was associated with improved PFS and showed a borderline association with OS, in line with the widely described “obesity paradox” observed in other immunotherapy-treated malignancies, including melanoma, lung and renal cell cancer, and MCC (Zhang et al. 2023; Xu et al. 2024; Incorvaia et al. 2023; Johannet et al. 2020; Krejčí et al. 2025). Strippoli et al. (2021), who evaluated a small cohort of cemiplimab-treated cSCC patients, did not detect such an association, which may relate to limited sample size and differences in population frailty. Several mechanisms have been proposed to explain improved outcomes in higher-BMI patients, including altered cytokine signaling, enhanced metabolic reserve supporting T-cell activation, and improved pharmacokinetic drug exposure. Given that advanced cSCC predominantly affects elderly patients, in whom nutritional status, frailty, sarcopenia, and cachexia strongly influence treatment tolerance and survival, BMI may partly act as a crude surrogate for physiologic reserve rather than a direct immunologic modifier. Accordingly, the observed BMI association should be interpreted cautiously and warrants prospective validation using standardized body-composition metrics.

Despite the exploratory associations observed for BMI and LMR, DSS remained overwhelmingly driven by AJCC stage IV, underscoring that tumor burden and metastatic pattern are the primary determinants of DSS in advanced cSCC. The low number of cSCC-specific events limited the complexity of multivariable DSS modelling. Inflammatory biomarkers showed no meaningful shifts between baseline and 3 months of cemiplimab therapy. This contrasts with some melanoma cohorts where early NLR or CRP changes correlated with outcome but is consistent with prior evidence that systemic inflammation in cSCC may stem from long-standing comorbidities or host immune aging rather than dynamic tumor–immune interactions (Chen et al. 2020; Bai et al. 2021; Rutkowski et al. 2018). This stability supports the use of baseline biomarkers rather than on-treatment changes for prediction in advanced cSCC.

This study has several limitations. Its retrospective multicenter design introduces potential selection and information bias, and imaging and follow-up schedules were not standardised across centers. Excluding patients with systemic immunosuppression improved biological interpretability of lymphocyte-based indices but reduces generalisability to this clinically relevant subgroup. Event numbers, particularly for OS and DSS, were modest and limited the complexity of multivariable modelling. Moreover, unmeasured host-related factors such as frailty, sarcopenia, nutritional status and unrecorded infections may have influenced both SIIBs and BMI. Prospective studies with standardised sampling, body-composition measures and integrated immunologic profiling are needed to refine the clinical utility of SIIBs in cSCC.

## Conclusions

Baseline SIIBs, particularly LMR, showed modest associations with treatment response but did not remain independent predictors after adjustment for clinical covariates. BMI demonstrated prognostic relevance for PFS and a borderline association with OS, while AJCC stage IV remained the dominant determinant of DSS. Comparison with an early-stage cohort revealed only modest stage-related shifts in SIIBs, suggesting that these markers reflect broad immune alterations rather than serving as reliable indicators of tumor progression or treatment outcomes within advanced-stage disease. Future prospective studies should evaluate SIIBs within composite biomarker frameworks and incorporate longitudinal sampling, body-composition metrics and microenvironmental profiling to refine risk stratification and inform individualised treatment approaches.

## Supplementary Information

Below is the link to the electronic supplementary material.

Supplementary Material 1

## Data Availability

The data that support the findings of this study are available from the corresponding author upon reasonable request.
