# Supplementary material for: Predictors of response and survival in cemiplimab–treated cutaneous squamous cell carcinoma: multicenter real-world evidence from Germany
Source: J Cancer Res Clin Oncol. 2026 Feb 25;152(2):50. doi: 10.1007/s00432-026-06423-x (PMC12936293; doi:10.1007/s00432-026-06423-x)
Supplement: Supplementary file 1 — Supplementary Material 1 [file 432_2026_6423_MOESM1_ESM.docx]

**Supplementary Tables**

**Supplementary Table S1A. Patient characteristics stratified by baseline LMR (median cut-off).**

Cut-off defined as the cohort median baseline LMR = 1.87.

| Parameter | Total (n=110) | LMR < 1.87 (n=55) | LMR ≥ 1.87 (n=55) |
| --- | --- | --- | --- |
| Age, median (range) | 84, 39-99 | 82, 58-99 | 86, 39-97 |
| Sex (f, m) | 36 (32.7%), 74 (67.3%) | 17 (30.9%), 38 (69.1%) | 19 (34.5%), 36 (65.5%) |
| BMI (mean±SD; median) | 25.6±4.7 (median 24.8) | 24.8±3.7 (median 24.4) | 26.4±5.5 (median 26.1) |
| CCI (mean±SD; median) | 8.2±2.6 (median 8.0) | 8.1±2.4 (median 8.0) | 8.3±2.7 (median 8.0) |
| Primary tumour location | Head/neck 77 (70.0%); Upper limbs 17 (15.5%); Lower limbs 6 (5.5%); Trunk 4 (3.6%); Genital/perianal 6 (5.5%) | Head/neck 35 (63.6%); Upper limbs 11 (20.0%); Lower limbs 3 (5.5%); Trunk 3 (5.5%); Genital/perianal 3 (5.5%) | Head/neck 42 (76.4%); Upper limbs 6 (10.9%); Lower limbs 3 (5.5%); Trunk 1 (1.8%); Genital/perianal 3 (5.5%) |
| Lower lip/ear involvement (yes) | 24 (21.8%) | 11 (20.0%) | 13 (23.6%) |
| Differentiation >= G3 (yes) | 21 (19.1%) | 9 (16.4%) | 12 (21.8%) |
| Thickness >6mm (yes) | 32 (29.1%) | 15 (27.3%) | 17 (30.9%) |
| Horizontal diameter >2cm (yes) | 65 (59.1%) | 34 (61.8%) | 31 (56.4%) |
| PNI/desmoplasia (yes) | 25 (22.7%) | 15 (27.3%) | 10 (18.2%) |
| AJCC stage (II/III/IV) | II 12 (10.9%); III 53 (48.2%); IV 45 (40.9%) | II 6 (10.9%); III 29 (52.7%); IV 20 (36.4%) | II 6 (10.9%); III 24 (43.6%); IV 25 (45.5%) |
| Radiotherapy to primary tumour (yes) | 43 (39.1%) | 25 (45.5%) | 18 (32.7%) |
| Number of ICI cycles (mean±SD; median) | 10.6±9.8 (median 9.0) | 9.7±7.1 (median 8.0) | 11.4±12.0 (median 9.0) |
| Baseline LMR, median (IQR) | 1.87 (1.34-2.68) | 1.33 (1.07-1.56) | 2.69 (2.20-4.21) |
| Baseline NLR, median (IQR) | 4.17 (2.60-6.26) | 6.00 (4.32-8.24) | 2.60 (2.14-3.85) |
| Baseline SIRI, median (IQR) | 2.90 (1.56-5.10) | 5.15 (3.22-6.81) | 1.63 (1.15-2.32) |
| ORR (yes) | 79 (71.8%) | 36 (65.5%) | 43 (78.2%) |
| PFS events (progression/death) | 62 (56.4%) | 32 (58.2%) | 30 (54.5%) |
| OS events (death) | 34 (30.9%) | 16 (29.1%) | 18 (32.7%) |
| cSCC-specific deaths | 11 (10.0%) | 4 (7.3%) | 7 (12.7%) |

**Supplementary Table S1B. Patient characteristics stratified by baseline SIRI (median cut-off).**

Cut-off defined as the cohort median baseline SIRI = 2.90.

| Parameter | Total (n=110) | SIRI < 2.90 (n=55) | SIRI ≥ 2.90 (n=55) |
| --- | --- | --- | --- |
| Age, median (range) | 84, 39-99 | 85, 39-97 | 83, 60-99 |
| Sex (f, m) | 36 (32.7%), 74 (67.3%) | 18 (32.7%), 37 (67.3%) | 18 (32.7%), 37 (67.3%) |
| BMI (mean±SD; median) | 25.6±4.7 (median 24.8) | 25.9±5.2 (median 25.6) | 25.3±4.3 (median 24.6) |
| CCI (mean±SD; median) | 8.2±2.6 (median 8.0) | 8.1±2.8 (median 8.0) | 8.2±2.3 (median 8.0) |
| Primary tumour location | Head/neck 77 (70.0%); Upper limbs 17 (15.5%); Lower limbs 6 (5.5%); Trunk 4 (3.6%); Genital/perianal 6 (5.5%) | Head/neck 42 (76.4%); Upper limbs 5 (9.1%); Lower limbs 3 (5.5%); Trunk 2 (3.6%); Genital/perianal 3 (5.5%) | Head/neck 35 (63.6%); Upper limbs 12 (21.8%); Lower limbs 3 (5.5%); Trunk 2 (3.6%); Genital/perianal 3 (5.5%) |
| Lower lip/ear involvement (yes) | 24 (21.8%) | 12 (21.8%) | 12 (21.8%) |
| Differentiation >= G3 (yes) | 21 (19.1%) | 12 (21.8%) | 9 (16.4%) |
| Thickness >6mm (yes) | 32 (29.1%) | 18 (32.7%) | 14 (25.5%) |
| Horizontal diameter >2cm (yes) | 65 (59.1%) | 29 (52.7%) | 36 (65.5%) |
| PNI/desmoplasia (yes) | 25 (22.7%) | 12 (21.8%) | 13 (23.6%) |
| AJCC stage (II/III/IV) | II 12 (10.9%); III 53 (48.2%); IV 45 (40.9%) | II 8 (14.5%); III 24 (43.6%); IV 23 (41.8%) | II 4 (7.3%); III 29 (52.7%); IV 22 (40.0%) |
| Radiotherapy to primary tumour (yes) | 43 (39.1%) | 17 (30.9%) | 26 (47.3%) |
| Number of ICI cycles (mean±SD; median) | 10.6±9.8 (median 9.0) | 11.4±11.8 (median 9.0) | 9.7±7.4 (median 8.0) |
| Baseline LMR, median (IQR) | 1.87 (1.34-2.68) | 2.69 (2.12-4.21) | 1.33 (1.07-1.60) |
| Baseline NLR, median (IQR) | 4.17 (2.60-6.26) | 2.60 (2.14-3.51) | 6.22 (4.75-8.81) |
| Baseline SIRI, median (IQR) | 2.90 (1.56-5.10) | 1.54 (1.15-2.25) | 5.15 (3.42-6.81) |
| ORR (yes) | 79 (71.8%) | 44 (80.0%) | 35 (63.6%) |
| PFS events (progression/death) | 62 (56.4%) | 30 (54.5%) | 32 (58.2%) |
| OS events (death) | 34 (30.9%) | 18 (32.7%) | 16 (29.1%) |
| cSCC-specific deaths | 11 (10.0%) | 6 (10.9%) | 5 (9.1%) |

Supplementary Table S1C. Patient characteristics stratified by baseline NLR (median cut-off).

Cut-off defined as the cohort median baseline NLR = 4.17.

| Parameter | Total (n=110) | NLR < 4.17 (n=55) | NLR ≥ 4.17 (n=55) |
| --- | --- | --- | --- |
| Age, median (range) | 84, 39-99 | 83, 39-94 | 84, 60-99 |
| Sex (f, m) | 36 (32.7%), 74 (67.3%) | 19 (34.5%), 36 (65.5%) | 17 (30.9%), 38 (69.1%) |
| BMI (mean±SD; median) | 25.6±4.7 (median 24.8) | 25.9±5.1 (median 25.8) | 25.3±4.4 (median 24.5) |
| CCI (mean±SD; median) | 8.2±2.6 (median 8.0) | 8.2±2.7 (median 8.0) | 8.1±2.4 (median 8.0) |
| Primary tumour location | Head/neck 77 (70.0%); Upper limbs 17 (15.5%); Lower limbs 6 (5.5%); Trunk 4 (3.6%); Genital/perianal 6 (5.5%) | Head/neck 41 (74.5%); Upper limbs 8 (14.5%); Lower limbs 2 (3.6%); Trunk 1 (1.8%); Genital/perianal 3 (5.5%) | Head/neck 36 (65.5%); Upper limbs 9 (16.4%); Lower limbs 4 (7.3%); Trunk 3 (5.5%); Genital/perianal 3 (5.5%) |
| Lower lip/ear involvement (yes) | 24 (21.8%) | 14 (25.5%) | 10 (18.2%) |
| Differentiation >= G3 (yes) | 21 (19.1%) | 12 (21.8%) | 9 (16.4%) |
| Thickness >6mm (yes) | 32 (29.1%) | 20 (36.4%) | 12 (21.8%) |
| Horizontal diameter >2cm (yes) | 65 (59.1%) | 29 (52.7%) | 36 (65.5%) |
| PNI/desmoplasia (yes) | 25 (22.7%) | 12 (21.8%) | 13 (23.6%) |
| AJCC stage (II/III/IV) | II 12 (10.9%); III 53 (48.2%); IV 45 (40.9%) | II 7 (12.7%); III 22 (40.0%); IV 26 (47.3%) | II 5 (9.1%); III 31 (56.4%); IV 19 (34.5%) |
| Radiotherapy to primary tumour (yes) | 43 (39.1%) | 17 (30.9%) | 26 (47.3%) |
| Number of ICI cycles (mean±SD; median) | 10.6±9.8 (median 9.0) | 11.3±11.7 (median 9.0) | 9.9±7.6 (median 8.0) |
| Baseline LMR, median (IQR) | 1.87 (1.34-2.68) | 2.58 (1.96-4.04) | 1.41 (1.14-1.76) |
| Baseline NLR, median (IQR) | 4.17 (2.60-6.26) | 2.60 (2.14-3.36) | 6.28 (5.17-9.08) |
| Baseline SIRI, median (IQR) | 2.90 (1.56-5.10) | 1.54 (1.15-2.28) | 4.72 (3.17-6.77) |
| ORR (yes) | 79 (71.8%) | 44 (80.0%) | 35 (63.6%) |
| PFS events (progression/death) | 62 (56.4%) | 31 (56.4%) | 31 (56.4%) |
| OS events (death) | 34 (30.9%) | 19 (34.5%) | 15 (27.3%) |
| cSCC-specific deaths | 11 (10.0%) | 7 (12.7%) | 4 (7.3%) |
